# Genetic Screening and Expression Analysis of Psychrophilic *Bacillus* spp. Reveal Their Potential to Alleviate Cold Stress and Modulate Phytohormones in Wheat

**DOI:** 10.3390/microorganisms7090337

**Published:** 2019-09-10

**Authors:** Muhammad Zubair, Alvina Hanif, Ayaz Farzand, Taha Majid Mahmood Sheikh, Abdur Rashid Khan, Muhammad Suleman, Muhammad Ayaz, Xuewen Gao

**Affiliations:** 1 Department of Plant Pathology, College of Plant Protection, Nanjing Agricultural University, Key Laboratory of Integrated Management of Crop Diseases and Pests, Ministry of Education, Nanjing 210095, China; zubair_biotech@yahoo.com (M.Z.); rao.alvina@yahoo.com (A.H.); ayaz.farzand@uaf.edu.pk (A.F.); tahamajid1705@yahoo.com (T.M.M.S.); malix.477@hotmail.com (A.R.K.); ayazbbt@yahoo.com (M.A.); 2 Department of Plant Pathology, University of Agriculture, Faisalabad P.O. Box 38040, Pakistan; 3 Institute of Microbiology, School of life sciences, Lanzhou University, Lanzhou 730000, China; suleman2017@lzu.edu.cn

**Keywords:** psychrophilic, PGPR, genetic screening, signal transduction, osmotic regulation, plant-stress response

## Abstract

Abiotic stress in plants pose a major threat to cereal crop production worldwide and cold stress is also notorious for causing a decrease in plant growth and yield in wheat. The present study was designed to alleviate cold stress on plants by inoculating psychrophilic PGPR bacteria belonging to Bacillus genera isolated from extreme rhizospheric environments of Qinghai-Tibetan plateau. The genetic screening of psychrophilic *Bacillus* spp. CJCL2, RJGP41 and temperate *B. velezensis* FZB42 revealed presence of genetic features corresponding to cold stress response, membrane transport, signal transduction and osmotic regulation. Subsequently, the time frame study for the expression of genes involved in these pathways was also significantly higher in psychrophilic strains as analyzed through qPCR analysis at 4 ℃. The inoculated cold tolerant Bacillus strains also aided in inducing stress response in wheat by regulating abscisic acid, lipid peroxidation and proline accumulation pathways in a beneficial manner. Moreover, during comparative analysis of growth promotion in wheat all three Bacillus strains showed significant results at 25 ℃. Whereas, psychrophilic Bacillus strains CJCL2 and RJGP41 were able to positively regulate the expression of phytohormones leading to significant improvement in plant growth under cold stress.

## 1. Introduction

The cereal crops are the basis of agricultural production and are staple food for more than half of the world’s population [1]. Being the major cereal crop, wheat is required to be produced in abundance to meet the steadily escalating demands of rapidly increasing world population. Abiotic stresses are reported to have detrimental effects on the production of the wheat crop worldwide [2]. Among abiotic stresses, cold stress is a major plant stress that can limit wheat crop production by inducing physiological and metabolic disparities leading to Reactive Oxygen Species ROS accumulation, nutritional disorders, membrane dysfunctioning, reduced photosynthetic ability and hormonal imbalance [3,4]. The plants have developed multiple defense mechanisms to combat such stress but still the losses in the productivity of staple food crops due to abiotic stresses has reportedly risen to almost 70% [5].

The application of Plant Growth Promoting Rhizobacteria (PGPR) has been widely reported to alleviate abiotic stress in plants [6]. PGPR can help to withstand cold stress by inducing anti-oxidant, hormonal, photosynthetic and other stress related pathways in plants [7,8]. Among PGPR, *Bacillus* strains are a spore-forming, gram positive, plant colonizing and growth promoting bacteria [9,10]. *Bacillus* are functionally diverse group of microbes which can produce a variety of metabolic products and possess the ability to withstand the harsh environmental conditions due to their natural stability and rigidness [11,12]. In this study we used psychrophilic (cold-loving) *Bacillus* spp. RJGP41 and CJCL2 isolated from the Qinghai-Tibet Plateau and a well reported biocontrol and plant growth promoting strain *Bacillus velezensis FZB42* [13] to evaluate their potential to alleviate cold stress in wheat. There are some reports to assess the beneficial effects of gram-negative [14] and gram positive bacteria [15] on plants under cold stress but there have been very rare reports providing any genomic or gene expression related insights to confirm such potential of bacteria.

The stress resistance capability of *Bacillus* is attributable to presence and expression of certain genetic features in their genome which enables them to express such physiological responses [16,17]. The gene families related to cold shock proteins [18], signal transduction pathway [19], oxidative stress and antioxidant enzymes [20], osmotic regulation [21] and membrane transportation [22] are widely accredited to the potential of *Bacillus* to withstand cold stress. The expression of these gene families results in certain transcriptional changes responsible for better water transport across their membrane, maintain water balance in the cell and better response to cold stress by regulation of important signaling pathways. Along with all these essential genetic features and their products, biofilm formation is also an important parameter for the *Bacillus* to colonize and produce diverse and important secondary metabolites under harsh environmental conditions [23].

Plant stress response parameters such as abscisic acid (ABA), lipid peroxidation and proline accumulation can regulate the stress adaptability in plants as these are indigenously produced signal molecules that can influence biological responses in plant locally or distally [24]. Some PGPR including *Bacillus* can modulate such stress parameters and influence stress adaptability in plants [25]. ABA has been reported to influence root growth and water content under abiotic conditions in plants [26] and the usual increased level of ABA under osmotic stress have been reported to be downregulated by inoculation of plant growth promoting PGP *Bacillus* in plants [27]. Malondialdehyde (MDA) is considered as a marker for oxidative lipid injury or peroxidation of membrane lipids in plants under abiotic stress [28] and PGP bacteria have been reported to decrease MDA levels in plants [29]. Proline accumulation in plants serve as an osmolyte and osmo-protectant; acting as a major factor in plant defense against environmental stress and PGPR *Bacillus* upon plant inoculation are able to influence proline accumulation in a positive manner [30]. 

Plant growth promotion by inoculated *Bacillus* spp. under different abiotic stress conditions has been widely reported; *Bacillus* can enhance plant growth directly by the virtue of possessing genes involved in plant growth promotion [31] and indirectly by regulating the plant stress response and phytohormone production in plants [32]. Presence of ACC deaminase gene in bacteria confers the ability to lower the ethylene levels under abiotic stress in plants resulting in plant growth promotion [33]. Under environmental stress, the up-regulation of plant growth promoting hormones such as auxin and cytokine under the influence of inoculated bacteria also results in improving the growth of wheat plants [34]. Expansins are considered to be the major regulators of cell wall extension and contribute to plant growth and development in stressed environments as well [35]. The plant growth promoting *Bacillus* strains can also stimulate the expression of expansins and ultimately contribute to plant growth promotion [36]. 

Therefore, aim of the present study was to carry out genetic screening and expression analysis for investigating the potential genetic and physiological features of *Bacillus* spp. which enable them to withstand cold stress and perform their metabolic functions as well. The *Bacillus* spp. able to do so could also alleviate the adverse effects of cold stress on plants; enhancing the growth of wheat plants under cold stress by regulation of stress responses and plant growth hormones.

## 2. Materials and Methods

### 2.1. Bacterial Growth Screening and Time-Frame Study for Cold-Adaptive Strains

The strains used in this study were isolated from rhizospheric soil of different Tibetan prefectures able to naturally survive at low temperature such as 4 °C. Two potential plant growth promoting cold tolerant *Bacillus* strains along with a non-tolerant PGP strain *Bacillus velezensis* were selected for their ability to grow at low temperature by inoculating the liquid culture of bacteria (in log phase) on LB agar plates and were kept at 25 °C, 14 °C, 10 °C, and 4 °C for up to 96 h. These bacteria were also examined for their ability to grow at low temperature at different time intervals i.e., 12 h, 24 h, 36 h, 48 h, 60 h, 72 h, 84 h and 96 h post inoculation in liquid culture. The growth pattern of cold tolerant strains (CJCL2 and RJGP41) and cold non-tolerant strain (FZB42) was determined by growing them at 25 °C, 14 °C, 10 °C, and 4 °C simultaneously. The growth was observed through O.D (at 600 nm) taken by using spectrophotometer at mentioned time intervals. The comparative growth curves were generated for all *Bacillus* strains in order to determine their growth pattern. The experiment was repeated in triplicate with three replicates for each treatment.

### 2.2. Extraction of Total Genomic DNA

For the isolation of total genomic DNA, PGPR bacteria CJCL2, RJGP41 and FZB42 were streaked on LB agar plates and incubated for 48–72 h at 30 ± 2 °C. Single bacterial colony was inoculated to LB broth medium (10 mL) and put for shaking at shaker (150 rpm) at 30 °C for 24 h. Bacterial culture (1 mL) was centrifuged at 13,000 rpm for 2 min to get pellet of cell. These bacterial cells were used for the extraction of total genomic DNA using DNA Extraction kit D3350-01 (Omega Bio-Tek, Norcross, GA, USA) according to manufacturer’s guidlines.

### 2.3. Detection of Genes Involved in Cold Tolerance

The candidate/unique genes were detected in the *Bacillus* strains already screened for their growth at low temperature i.e, 4 °C [17]. Each of the reaction mixture of PCR was 25 µL which contained 12.5 µL of DNA master mix (Vazyme Biotech. Co. ltd, Nanjing, China), forward and reverse primers (1 µL each) for all the genes under screening, 1 µL DNA template and 9.5 µL ddH_2_O. The PCR profile was as: initial denaturation at 95 °C for 3 min, then 32 cycles of: denaturation for 15 s at 95 °C, annealing at 55 °C for 15 s, extension for 15 s at 72 °C and the final extension at 72 °C for 3 min. A total of 1% agarose gel in 1 × TBE buffer was used to separate amplified PCR products of various gene sizes. A 2000 DL DNA ladder was used as maker for band size. Gel was observed under UV light and was photographed using gel documentation system. The PCR primers being used in this study were designed using PrimerQuest tool from IDT and are given in Table S1.

### 2.4. RNA Extraction and cDNA Synthesis

RNA was extracted from the bacterial strains (CJCL2, RJGP41 and FZB42) after growing them in LB media at low temperature (4 °C) and optimum temperature for the bacterial growth (37 °C) in a shaking incubator. The samples were harvested at different time intervals i.e., 24 h, 48 h, 72 h and 96 h post-inoculation to extract RNA in order to observe the expression of various genes said to be involved in cold stress resistance in bacteria. The RNA was extracted by following the protocol given by Bacterial RNA extraction kit (OMEGA Bio-tek, Inc. Norcross, GA, USA). The concentration and purity of isolated RNA was determined by measuring the absorbance at 260/280 nm (NanoDrop 1000, Thermo Scientific, Wilmington, DE, USA). 

Extracted bacterial RNA samples at different time intervals were used for the synthesizing cDNA in order to perform qPCR analysis of cold tolerant as well as non-cold tolerant bacteria. For cDNA synthesis, 5 × ALL-In-One RT MasterMix (with AccuRT Genomic DNA Removal Kit) by Applied Biological Materials Inc. (abm^®^, Beijing) was used and reaction mixture and PCR conditions were followed as mentioned in the protocol given with the kit.

### 2.5. Expression Profiling of Bacterial Genes Influencing Cold Stress Tolerance by qPCR

The RT-qPCR was carried out in a Step One Real-Time PCR System (Applied Bio Systems, Foster City, CA, USA) based on the changes in fluorescence relative to the cyclic increase in the PCR products. The detector used was the SYBR Green (Takara Bio, Beijing, China), which emits fluorescence while binding to cDNA. The fluorescence value was recorded at the threshold cycle (C). The primers were designed using Primer Quest tool of IDT and these primers for RT-qPCR for cold-tolerant genes are listed in the Table S2. The *rpsJ* gene already reported as a house keeping gene was used as the endogenous control for *Bacillus* strains under study [37]. Real time PCR was carried out in 20 µL reaction volume containing 10 µL 2X SYBR premix Ex Taq (Takara Bio, Beijing, China)(Til RNaseH Plus) with Rox as a reference dye, 0.4 µL forward and reverse primers (20 nmol), 2 µL cDNA (100 ng), and 6.8 µL ddH_2_O. the expression was recorded by using initial denaturation at 95 °C for 30 s, including 40 cycles of 95 °C for 5 s, and 34 s at 60 °C was used. The accuracy of the reactions was analyzed using the T melt curve analysis at the end of PCR program. The final relative quantification was done based on the comparative C method of 2^−ΔΔ*C*T^ as explained by [38]. 

### 2.6. ROS Production in Bacillus spp. Under Cold Stress

Reactive Oxygen Species (ROS) are associated directly with any cellular disorder or dysfunction as a result of stress. ROS were tested in microbes grown under cold stress as a measure of disturbance in regular cell functions. For this purpose, the *Bacillus* strains, i.e., FZB42, CJCL2, and RJGP41 were cultured at 4 °C overnight and the cells were harvested by centrifugation at 96 h post-inoculation. The harvested cells were incubated for 30 min at 25 °C in 1.5 mL Eppendorf tubes containing a mixture of 10 Mm sodium phosphate buffer with pH 7.4 and dichloro-dihydro-fluoresein diacetate (DCFH-DA) (JianCheng Bioengineering, Nanjing, China) [39]. This dye can stain the sample containing ROS and green fluorescence was observed with the help of fluorescent microscopy using Olympus1X71 microscope and Image Pro express software v.6.2 (Olympus, Tokyo, Japan).

### 2.7. Bacterial Biofilm Formation Studies Under Cold Stress

Biofilm formation is a major characteristic of microorganisms by which they can attach to surfaces and perform various functions in their community structure. To test whether the cold stress had any adverse effects on biofilm formation of PGPR *Bacillus* bacteria, a novel biofilm-promoting medium (LBGM: LB plus 1% (vol/v glycerol and 0.1 mM MnSO4) was used [40]. The cold tolerant strains, i.e., CJCL2, RJGP41, and cold non-tolerant *Bacillus* FZB42 were shaken in 20 mL LB broth at 37 °C incubator to attain an O.D. of 1.0. Four microliter of bacterial cultures were then mixed with 4 mL of LBGM medium and the resultant mixtures were poured into costar^®^ Sterile 12 well cell culture cluster plates. The plates were tightly sealed and further incubated for up to 96 h at 37 °C and 4 °C The effect of cold stress on bacterial biofilm formation was observed in 12 well plates as well as by using Confocal laser scanning microscope (Confocal Microscope Zeiss LSM 780, Japan).

### 2.8. Molecular detection of PGP traits in Bacteria

Three major genes involved in imparting Plant Growth Promoting (PGP) potential to bacteria were screened by using gene specific primers. The *acdS* gene, encoding ACC deaminase enzyme is involved in promoting plant growth by lowering plant ethylene levels. To obtain the complete *acdS* gene sequences (1017 bp), a set of degenerate primers were used as previously described by [41]. The *gdh* gene (740 bp) encoding for glucose dehydrogenase was also detected by using gene specific primers complimentary to the gene sequences present in genome of *Bacillus* strains designed by Primer Quest tool of IDT [42]. The *pqqE* (451 bp) gene encoding for major PQQ cofactor involved in phosphate solubilization potential of these *Bacillus* strain was mapped by using degenerate primers of most conserved region in *pqq* operon as reported by [43]. All the primers are listed in Table S1.

### 2.9. Effect of Cold stress on Vigour Index of Wheat seedlings

The impact of cold stress on wheat seed-germination was studied by measuring the vigor index under cold stress (14 °C) and at regular growth temperature (25 °C) [20]. Seeds of wheat were surface sterilized with 5% sodium hypochlorite followed by washing with 70% ethanol and autoclaved distilled water. The sterilized seeds were inoculated with the cold tolerant *Bacillus* strains, i.e., CJCL2, RJGP41, and cold non-tolerant PGPR bacteria FZB42 separately and were incubated at 14 ± 2 °C and 25 ± 2 °C in three replicated Petri dishes (9 cm diameter) containing 0.3% water agar. Un-inoculated seeds dipped in sterilized water were used as control and were also kept on water agar plates.

The germination was observed when the radicals were half of the seed length. Percent (%) germination was recorded at 48 h and 72 h post inoculation. The seedling Root and shoot length were measured after seven days. The experiment was carried out as a completely randomized design (CRD) with 3 plates of three seeds for each bacterium. The % germination and vigour index were measured using formula reported by [44].

### 2.10. Seedling Root morphology studies under cold stress

The cold stress tolerance ability of wheat seedling was tested when PGPR inoculated wheat seedlings were subjected to root morphological studies. The wheat seeds were first surface sterilized by using 5% sodium hypochlorite solution followed by washing with 70% ethanol. The seeds were then washed three times with ddH2O. The wheat seeds were inoculated with PGPR *Bacillus* and grown on MS media at 14 ± 2 °C and 25 ± 2 °C to analyze the cold stress tolerance potential of inoculated *Bacillus*. The control had wheat seeds only soaked in LB media without inoculation of any bacteria.

For determining the root morphology, the wheat seedlings were observed after 7 days. All seedlings were selected in three replicates for different root parameters. Root length, surface area, volume, diameter, and number of root tips of each seedling were determined using Rhizoscanner (EPSON Perfection V700 Photo, Epson America, Long Beach, CA, USA), equipped with WinRHIZO software offered by Regent Instruments Co (Sainte-Foy, Quebec, Canada) [44].

### 2.11. Quantification and Expression Profiling of Plant Stress Response Parameters

The wheat plants were analyzed for parameters responsible for inducing stress response. The treated and control wheat samples grown under cold stress i.e., 4 °C and optimum temperature i.e., 25 °C were analyzed for abscisic acid, lipid peroxidation and proline accumulation. The leaves were harvested randomly in three replicates from three pots of each treatment after 9 days Post Inoculation (dpi) of wheat plants transplanted from seedlings already grown for 7 days.

#### 2.11.1. Abscisic Acid

For quantification of abscisic acid (ABA), 0.1 g of leaves (FW) from each treatment were ground into a fine powder by adding liquid nitrogen and using a mortar and pestle and then the samples were individually transferred into 2 mL plastic Eppendorf tubes. The leaves were then homogenized in 80% methanol and the samples were kept at 300 rpm at 4 °C for overnight for extraction [45]. The samples were then vortexed and centrifuged at 14,000 rpm for 30 min at 4 °C. The supernatant was filtered and then dried by vacuumed evaporation for approximately 4 h at room temperature. The extract was then dissolved in 200 µL of 80% methanol and the samples was run on UPLC for ABA detection and quantification [46].

#### 2.11.2. Lipid Peroxidation

The lipid peroxidation (LP) level of control (un-inoculated) and treated (inoculated) tissues was determined by measuring malondialdehyde (MDA) content via 2-thiobarbituric acid (TBA) reaction using modified protocol described by [47]. Around 100 mg of wheat leaves were homogenized in 500 µL of 0.1% (*w/v*) TCA and centrifuged for 10 min at 13,000 *g* at 4C. Then 1.5 mL 0.5% TBA was mixed with 500 µL of supernatant and incubated in water bath at 95 °C for 25 min. This mixture was further incubated on ice for 5 min for the termination of ongoing reaction. The mixture was analyzed for absorbance at 532 and 600 nm in a microplate reader (Spectrum max plus; Molecular devices, Sunnyvale, CA, USA).

#### 2.11.3. Proline Accumulation

The proline was quantified using ethanolic extract prepared by homogenizing 100 mg fresh leaves in 1 mL of 70% ethanol [48]. The homogenized leaf mixture contained 1% *w/v* ninhydrin in 60% *v/v* acetic acid and 20% *v/v* ethanol, mixed with ethanolic extract in the ratio of 2:1. The reaction mixture quantified upto 100 µL was then incubated in a water bath at 95 °C for 20 min, then cooled to room temperature and absorbance was observed at 520 nm in a microplate reader (Spectrum max plus; Molecular devices, Sunnyvale, CA, USA).

#### 2.11.4. Expression Analysis of Stress Responsive Genes in Plants

Total RNA was extracted from wheat leaves harvested at 9 dpi from each treatment grown under cold stress as well as at regular temperature by using plant RNA extraction kit by following manufacturer’s protocol. The concentration and purity of the isolated RNA was verified by measuring its absorbance at 260/280 nm. The cDNA first strand was synthesized using 5X All-In-One Rt MasterMix with AccuRT genomic DNA Removal Kit (ABM). The synthesized cDNA was used as template for qRT-PCR.

### 2.12. Effect of Bacterial Isolates on Photosynthetic Potential of Plants Under Cold Stress

Photosynthesis rate and stomatal conductance of wheat plants grown at 25 °C and grown under cold stress was analyzed by taking readings from five maximum light exposed leaves from three plants of each treatment by using an open IRGA LI-COR 6400 XT portable photosynthesis system (LI-6400, Li-Cor Inc., Lincoln, NE, USA). Net photosynthetic rate (Pn) and stomatal conductance (gs) were noted under light saturated conditions at photosynthetic photon flux density of 1000 µmol photons m^−2^ s^−1^ and 380 mol mol^−1^ CO_2_ concentration [49].

### 2.13. In Planta Growth Promotion Under Cold Stress by Inoculated Bacteria

Plant growth promoting potential of cold tolerant and non-tolerant *Bacillus* strains was evaluated In Vivo in a pot experiment in growth chambers with low temperature condition i.e., 4 °C and at optimum growth environment i.e., 25 °C. Four treatments were considered with one being un-inoculated control and three selected bacteria included two cold tolerant strains CJCL2, RJGP41 and one cold non-tolerant biocontrol strain FZB42. The seedlings which were already grown for 7 days at 14 °C as mentioned in the previous section were transplanted into pots. All of these treatments were evaluated at both temperature conditions with three pots of three wheat seedlings each for every treatment. The plant fresh/dry weight was measured for each treatment 9 days post inoculation and the data was used as an indicator of plant growth promotion.

### 2.14. Plant Root Morphological Studies by Rhizoscanning

The plant root morphology studies were carried out by selecting three plants in each replicate randomly and parameters such as root length, surface area, volume, diameter and number of root tips of each treatment were analyzed by using root automatism scan apparatus Rhizoscanner, equipped with WinRHIZO software offered by Regent Instruments Co. The average values of these three plants were taken as one replicate. The wheat plant samples were taken 9 days post inoculation from the same experiment described in previous section; at low as well as optimum temperature. 

### 2.15. Expression Profiling of Growth Related Genes in Plants Under Cold Stress

For analyzing the expression of genes involved in plant growth promotion, the leaves were harvested from each treatment under cold stress and from regular temperature at 9 dpi from pots of plants who’s seedling has already been grown for 7 days. Total RNA extraction from leaves and cDNA synthesize was done as describes in previous section.

### 2.16. Quantitative PCR for Expression Studies in Plants

The sequences of the genes for plant stress response i.e., proline accumulation (*P5CS*), Lipid peroxidation (*4-HNE*) and abscisic acid (*ABARE*) and the genes involved in plant growth promotion (*expA1, CKX2, ERF* and *ARF*) were obtained from NCBI Genbank and the primers used for the relative quantification of biosynthetic gene transcripts were designed by using Primer Quest tool of Integrated DNA Technologies, The primers are listed in Table S2. The qRT-PCR analysis was carried out in a QuantStudio Real-Time Thermocycler (Thermo Fisher Scientific, San Jose, CA, USA) using chamQ SYBR green qRT-PCR Master mix (Vazyme Co. Ltd., Nanjing, China). The conditions used for qPCR were: Initial denaturation at 95 °C for 30 s, 40 cycles of 95 °C for 10 s and 30 s at 60 °C. The expression of genes under study was computed by using threshold (Ct) value for each gene normalized against the Ct for actin from wheat which was used as the constitutive reference transcript [50].

### 2.17. Statistical Analysis

All the in vitro and In planta experiments in this study were conducted in completely randomized and repeated thrice. Data was subjected to statistical analysis using statistical package SPSS. Means were separated using Tukey’s HSD at *p* ≤ 0.05 after ANOVA.

## 3. Results

### 3.1. Bacterial Growth Screening and Time-Frame Study for COLD-Adaptive Strains

**Figure 1:**
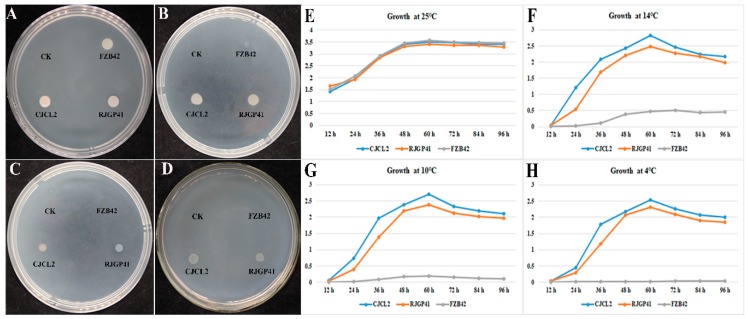
The colony growth of *Bacillus* strains grown on LB agar media and incubated at 25 °C for 96 h (**A**), at 14 °C for 96 h (**B**), at 10 °C (**C**), and at 4 °C (**D**). The growth curves indicated by OD_600_ of selected bacteria at 25 °C (**E**), 14 °C (**F**), 10 °C (**G**), and 4 °C (**H**) as measured by spectrophotometer at different time intervals. The experiment was repeated thrice with similar results.

The two bacterial strains *Bacillus* spp. CJCL2 and RJGP41 isolated from Qinghai-Tibetan plateau were able to grow under cold stress at 14 °C, 10 °C and 4 °C after 96 h post inoculation whereas the temperate *B. velezensis* strain FZB42 showed slight growth at 14 °C and was not able to grow on LB media at 4 °C (Figure 1). All three strains grew well at 25 °C.

The growth pattern of these three *Bacillus* strains was observed at various time intervals and the resulting growth curves indicated that growth of CJCL2 was increasing in a linear manner up to 96 h post-inoculation at 14 °C, 10 °C, and 4 °C (Figure 1). The cold tolerant strain CJCL2 grew best at low temperature followed by strain RJGP41 whereas the growth curve of FZB42 showed no significant increase at 14 °C, 10 °C, or 4 °C even up to 96 h post-inoculation. In contrast, all these strains grew well at regular temperatures, i.e., 25 and 37 °C.

### 3.2. Detection of Genes Involved in Cold Tolerance 

**Figure 2:**
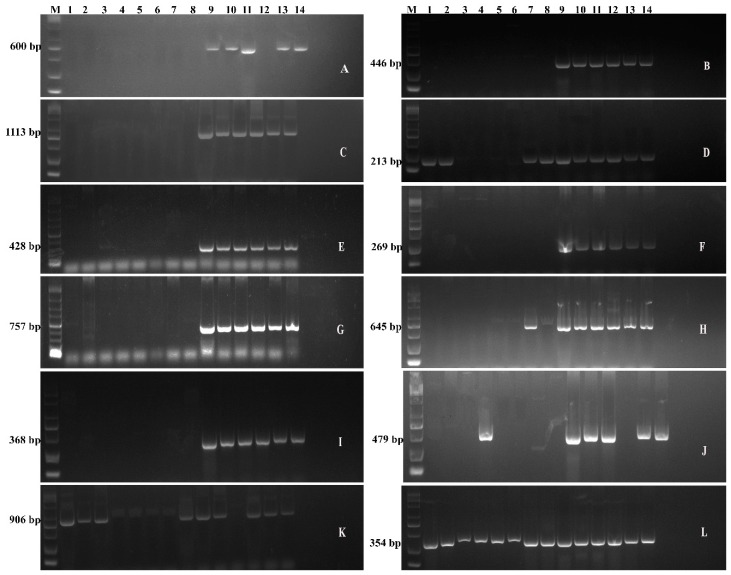
The Gel electrophoresis showing the detection of genes related to cold-tolerance in different *Bacillus* i.e., 1-GBSW11, 2-BS168, 3-LLCG23, 4-GBAC46, 5-DJFZ40, 6-LLTC96, 7-GBSW2, 8-FZB42, 9-CJCL2, 10-NMSW12, 11-RJGP41, 12-GBSW19, 13- LNXM10, 14-NMSL88 Whereas The genes detected are: *desR* (**A**), *SodA* (**B**), *desK* (**C**), *trxA* (**D**), *ResD* (**E**), *ohrR* (**F**), *DegS* (**G**), *ComA* (**H**), *dpsU20* (**I**), *OpuAC* (**J**), *KatA* (**K**), *perR* (**L**).

The unique genes thought to be involved in imparting cold-stress tolerance in *Bacillus* bacteria were predicted through comparative genomic analysis. The genes involved in signal transduction pathways, antioxidants, osmotic regulation, and sugar-abc transporters were screened in this study. All of these genes are directly involved in imparting cold stress tolerance to the bacteria. The PCR amplification products of these predicted candidate genes for cold-stress tolerance showed the majority of the genes were detected in the bacteria which are able to grow at low temperature, i.e., 4 °C such as CJCL2, RJGP41, GBSW19, LNXM10, and NMSL88 (Figure 2). The amplified products were sequenced and confirmed by using BLAST.

The genes involved in the two-component signal transduction pathway of bacteria, i.e., *desK* and *desR* by which bacteria senses and responds to certain stress, were detected in strains CJCL2, RJGP41, GBSW19, LNXM10, and NMSL88. These bacteria can tolerate low temperature. The low temperature increases the accumulation of reactive oxygen species and improves the stability of Oxy-radicals. Therefore, the presence of antioxidant genes is considered an important factor to combat cold stress. *DPSU20* is a 368 bp Oxidative damage protectant gene which was detected only in the strains which can withstand cold temperature. Whereas *KatA* was absent in cold tolerant bacteria RJGP41, i.e., sample no. 10, and was detected in some bacteria which cannot grow at low temperature. The 354 bp Peroxidase genes was amplified and detected in all the bacteria under study. The amplification of 446 bp Superoxide Dismutase gene was detected in all the cold tolerant bacteria, i.e., sample no. 9–14. In addition to cold tolerant bacteria, Thioredoxin gene *TrxA* was also detected in sample no. 2 and 3, i.e., NMSW12 and LLCG23, respectively. Organic hyperoxide gene *OhrR* was also detected in all cold-tolerant bacteria. The glycine betaine gene responsible for osmo-protection or osmotic regulation *OpuAC* was detected in almost all the cold-tolerant bacteria. *OpuAC* was also present in all the cold tolerant bacteria except GBSW19 and was additionally present in GBAC46. The PCR detection and amplification of predicted cold-stress tolerant genes showed that five bacteria, i.e., 9 to 14, are well-equipped with the genes that could be involved in imparting cold stress tolerance to the bacteria.

### 3.3. Expression Profiling of Genes Influencing Cold Stress Tolerance by Q-PCR

**Figure 3:**
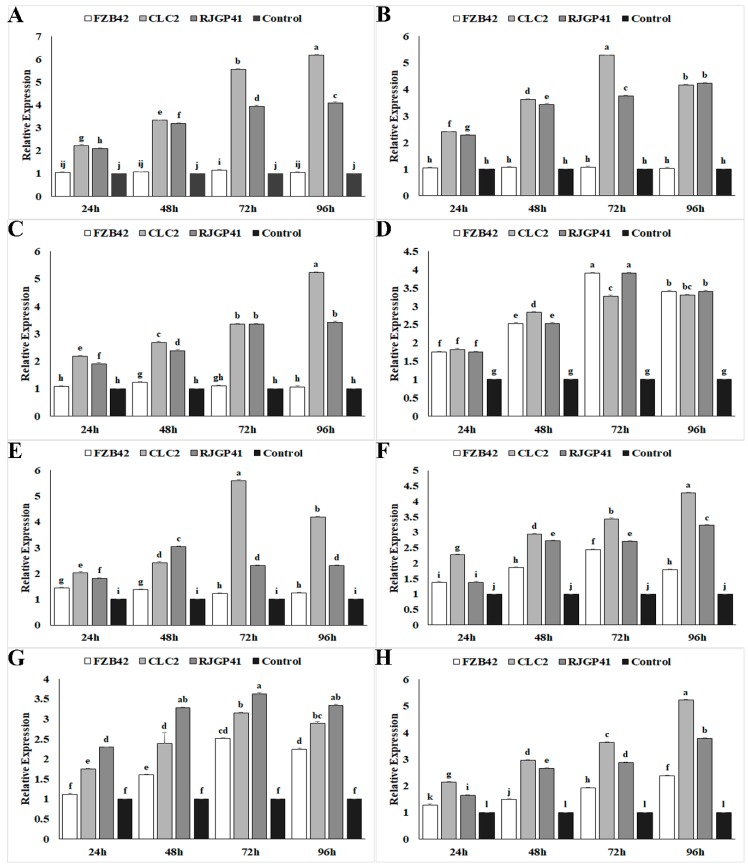
The graphs representing relative expression levels of different genes analyzed at multiple time intervals in *Bacillus* strains FZB42, CJCL2, and RJGP41 grown at 4 °C and 37 °C. The analyzed genes were: *cspB* (**A**), *cspC* (**B**), *cspD* (**C**), *comA* (**D**), *desK* (**E**), *desR* (**F**), *ohrR* (**G**), *OpuAC* (**H**). The error bars on the graphs indicate the standard deviation of the mean (*n* = 3). Letters above the columns represent significant differences between treatments at *p* ≤ 0.05. The expression studies were repeated three times with similar changes in expression.

The level of transcriptional regulation in genes responsible for expressing cold shock proteins (*CspB*, *CspC*, *CspD*) showed a linear increase in strains CJCL2 and RJGP41 up to 96 h post-inoculation at 4 °C (Figure 3). The strain CJCL2 showed maximum increase in expression levels of cold shock proteins with an upsurge of four–five-fold in expression as compared to the housekeeping control gene, whereas strain FZB42 did not show any noticeable increase in expression at any time interval. The genes related to osmotic regulation (*OhrR*), response quorum sensing regulator (*ComA*) also showed an increase in expression up to 72 h post-inoculation in CJCL2 and RJGP41 and the expression levels decreased slightly at 96 h. FZB42 also showed one–two-fold increase in expression of these genes at 96 h post-inoculation. The time-frame expression profiling of genes responsible for two-component signal transduction pathway combating abiotic stress (*DesK* and *DesR*) also showed an increase in expression levels of strains CJCL2 and RJGP41. The two-component system (*DesK* and *DesR*) genes showed maximum expression at 72 h in CJCL2 and at 96 h post-inoculation in RJGP41. The glycine betaine gene *OpuAC* showed maximum up-regulation at 96 h in CJCL2 strain. It showed a similarly high expression level at 72 and 96 h in RJGP41. All of these genes showed a very slight difference in expression levels as compared to control at 4 °C.

### 3.4. ROS Production in Bacillus spp. Under Cold Stress

**Figure 4:**
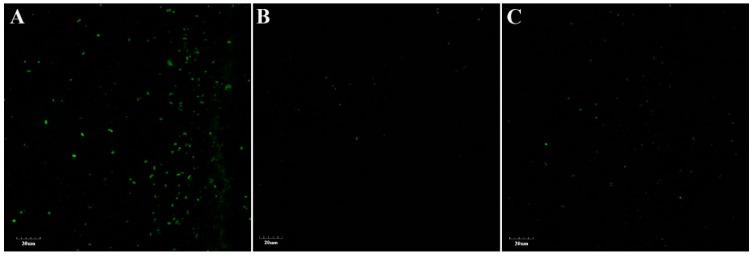
The higher level of green fluorescence indicating the higher level of Reactive Oxygen Species (ROS) in FZB42 strain grown for 96 h at 4 °C (**A**). The lower fluorescence depicts lesser ROS production in the cells of strain CJCL2 (**B**). The level of ROS as shown by fluorescent cells in strain RJGP41 (**C**).

The levels of reactive oxygen species in *Bacillus* strains were studied by culturing the bacteria at lower temperature, i.e., 4 °C. The results indicated by level of ROS stained cells as observed by CLSM that cold-tolerant strains CJCL2 and RJGP41 maintained lower levels of ROS at 96 h post-inoculation at 4 °C (Figure 4), whereas the strain FZB42 showed significant increase in the level of ROS at 96 h post-inoculation at 4 °C.

### 3.5. Bacterial Biofilm Formation Studies Under Cold Stress

**Figure 5:**
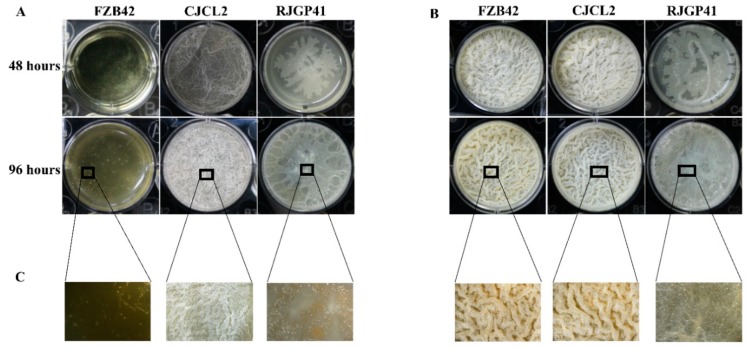
The biofilm formation potential of selected strains grown under cold stress (4 °C) (**A**). Biofilm formation ability of bacterial strains at optimum temperature, i.e., 37 °C (**B**). The 20 × images of biofilm structures of selected bacteria grown under cold stress as well as at optimum temperature (**C**).

The results of biofilm formation potential of all three strains at regular and lower temperature at two-time intervals (48 h and 96 h) showed that strain CJCL2 produced finest biofilm structure during cold stress at 48 h and 96 h post-inoculation (Figure 5). The strain RJGP41 also managed to make slight biofilm at 48 h and formed weekend biofilm that covered the whole well surface at 96 h post-inoculation at 4 °C. FZB42 failed to synthesize noticeable biofilm structure even at 96 h post-inoculation but having said that the finest biofilm structure was formed in case of FZB42 and CJCL2 at regular temperature, i.e., 37 °C.

### 3.6. Molecular detection of PGP traits in Bacteria

**Figure 6:**
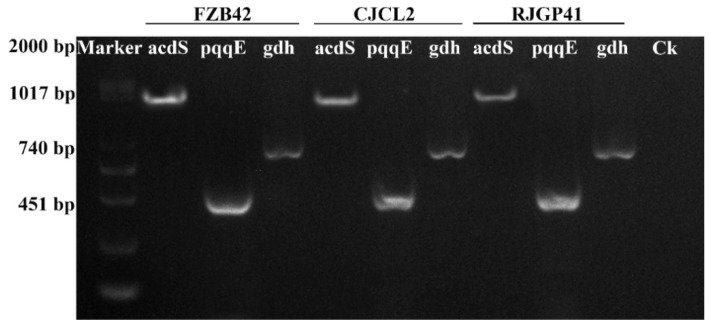
The Gel electrophoresis showing the detection of genes related to plant growth promoting traits in different bacteria.

The PCR based molecular detection of bacterial genes involved in direct or indirect promotion of plant growth showed that all three bacterial strains FZB42, CJCL2, and RJGP41 possessed these genes. The PCR amplification showed the presence of 1017 bp *acdS* gene encoding ACC Deaminase enzyme in all three strains (Figure 6). The 740 bp *gdh* encoding glucose dehydrogenase and 451 bp *pqqE* gene involved in phosphate solubilization were also detected in all three bacterial strains. The presence of all three genes showed the plant growth promoting potential of the *Bacillus* spp. under study.

### 3.7. Improvment in Seedling Growth and Root Morphological Parameters Under Cold Stress

**Figure 7:**
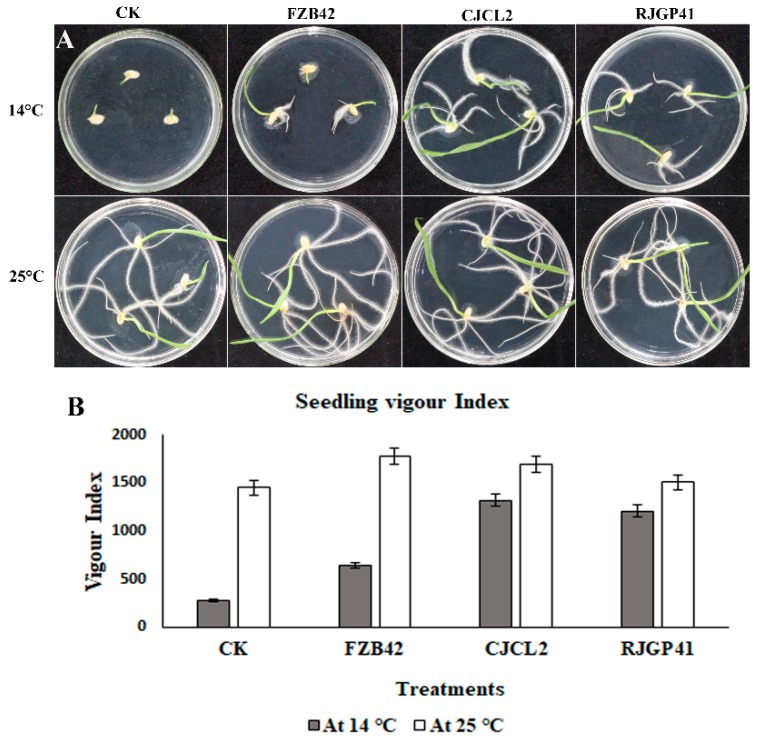
The wheat seeds grown for 7 days at 14 °C and 25 °C upon inoculation with strains FZB42, CJCL2, and RJGP41 (**A**). The measure of seedling length and percent germination given as a graph of seedling vigor index of inoculated seeds in comparison to control (**B**).

The inoculated *Bacillus* strains improved the seedling growth as well as root morphological parameters under cold stress. Seedling vigor index (VI) which is a measure of % germination and total length of seedling was observed to be maximum under cold stress in wheat seedlings treated with culture of CJCL2 (VI = 1720), which is significantly higher than the control seedlings (Figure 7). The strain RJGP41 gave the second highest VI in seedlings grown at low temperature (14 °C). In the wheat seedlings grown at regular temperature (25 °C), the seeds inoculated with FZB42 gave the best results for seedling growth and vigor index of the seedlings with a VI value of 1780 as compared to 1450 of un-inoculated control seedlings.

**Figure 8:**
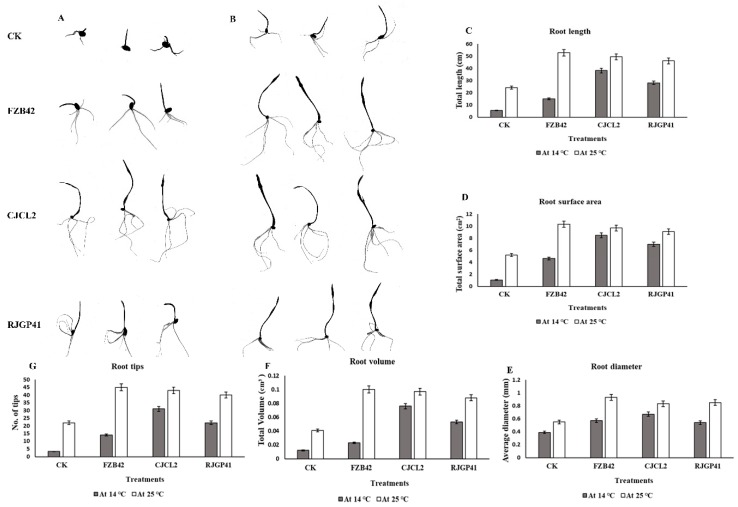
The rhizoscanning image of roots of seedling grown at 14 °C (**A**). The rhizoscanning image of roots of seedling grown at 25 °C (**B**). Root morphology parameters such as total root length (cm) of wheat seedling inoculated with selected *Bacillus* strains and grown at 14 and 25 °C (**C**). The total surface area (cm^2^) of roots in wheat seedlings (**D**), the diameter of the roots (mm) (**E**), the total root volume (cm^3^) (**F**). Number of root tips for each treatment (**G**) is comparatively represented by graphs. The error bars on the graphs indicate the standard error of the means. The significant difference among the treatments was observed by using Tukey’s HSD test at *p* ≤ 0.05. The experiment was repeated three times with similar results.

The significant difference among the treatments was observed by using Tukey’s HSD test at *p* ≤ 0.05 and the experiment was repeated in triplicate. The root morphological studies of wheat seedlings under cold stress and regular temperature demonstrated that the parameters such as root length, root diameter, root volume, root surface area and number of tips were found to be maximum in case of seedlings inoculated with CJCL2 under cold stress as compared to un-inoculated seedlings grown at 14 °C (Figure 8). The wheat seedlings inoculated with RJGP41 also showed prominent results for above mentioned parameters. In comparison, the seedlings inoculated with all three bacterial strains showed significant improvement for morphological parameters of roots as compared to control at regular temperature, i.e., 25 °C.

### 3.8. Quantification and Expression Profiling of Plant Stress Response Parameters

#### 3.8.1. Abscisic Acid

**Figure 9:**
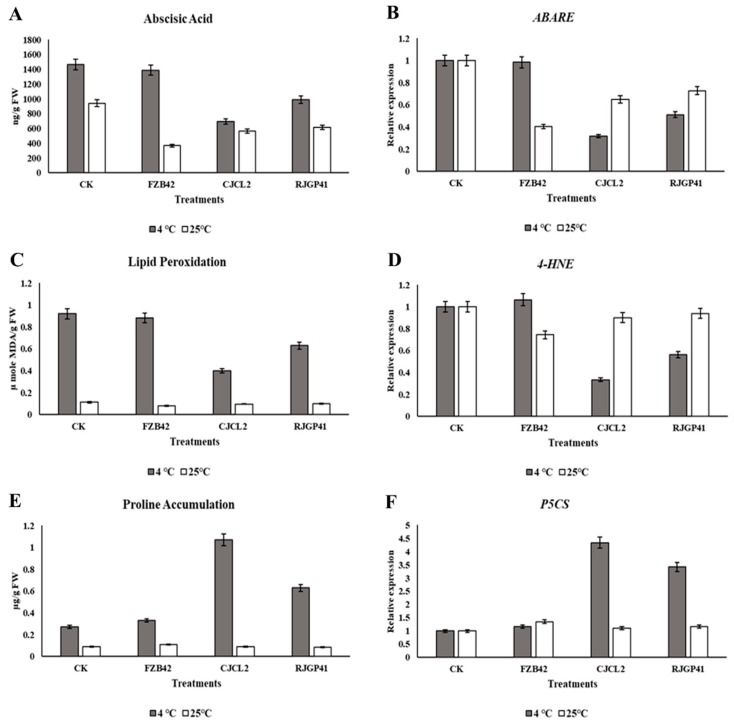
Quantification of abscisic acid by UPLC in wheat plants grown at 4 °C and 25 °C under different bacterial treatments (**A**). Relative expression level abscisic acid (ABA) encoding gene (*ABARE*) (**B**). Spectrophotometric quantification of malondialdehyde (MDA) from wheat plants (**C**). Relative expression level of MDA encoding gene (*4-HNE*) (**D**). Spectrophotometric quantification of proline (**E**). Relative expression level of *ARF* gene encoding proline synthesis (**F**). The error bars represent the standard deviation of the mean (*n* = 3). The significant difference was observed for all treatments by using Tukey’s HSD test at *p* ≤ 0.05 and the experiment was repeated in triplicate with similar results.

Quantification was done by UPLC analysis showed that the plants grown with *Bacillus* sp. CJCL2 treatment significantly reduced the ABA level in wheat plants under cold stress as compared to untreated control. The quantified value of ABA in CJCL2 treated plants was 692 ng/g FW as compared to 1465 ng/g FW in control plants (Figure 9). At regular growth temperature of wheat, FZB42 strain suppressed the ABA level most significantly i.e., 366 ng/g FW as compared to 940 ng/g FW of ABA in control. The other inoculated bacteria CJCl2 and RJGP41 also reduced the ABA levels in wheat plants grown at 25 °C.

#### 3.8.2. Lipid peroxidation

It was measured by content of Malondialdehyde (MDA) content by spectrophotometric values indicated level in inoculated and un-inoculated plants under cold stress as well as at regular temperature. The level of MDA was observed to be minimum in case of plants inoculated with CJCL2 (Figure 9). The inoculated *Bacillus* strains resulted in the reduction of MDA i.e., 0.473 µ mole MDA/g FW as compared to 0.926 µ mole MDA/g FW in control plants grown at 4 °C. The MDA content in FZB42 treated plants at low temperature didn’t show any significant decrease as compared to control plants. Whereas, the wheat plants grown at regular temperature showed normal levels of MDA for all treatments.

#### 3.8.3. Proline Accumulation

The present study in wheat plants showed that the proline content was observed to be increased significantly in plants treated with CJCL2 and RJGP41 under cold stress. The proline content in plants treated with *Bacillus* sp. CJCL2 showed significant enhancement in the proline content values i.e., 1.072 µg/g FW as compared to 0.273 µg/g FW of control under cold stress (Figure 9). The measured proline content for all treatments at optimum growth temperature (25 °C) showed no significant increase as compared to their control.

#### 3.8.4. Expression Profiling 

The expression profiling of genes involved in plant stress response showed significant changes in transcriptional regulations. Under cold stress, significant decrease in the expression levels of abscisic acid (ABA) and lipid peroxidation encoding genes *ABARE* and *4-HNE* was detected in plants inoculated with *Bacillus* sp. CJCL2 (Figure 9). There was a four fold increase in the expression of gene involved in Proline synthesis (*P5CS*) in the plants inoculated with same *Bacillus* grown under cold stress. There was no significant change in the expression of *4-HNE* or *P5CS* genes in different treatments of plants grown at optimum temperature i.e., 25 °C. Whereas, the expression of *ABARE* gene in plants grown at optimum temperature treated with FZB42 strain was significantly reduced.

### 3.9. Effect of Bacterial Isolates on Photosynthetic Potential of Plants Under Cold Stress

**Figure 10:**
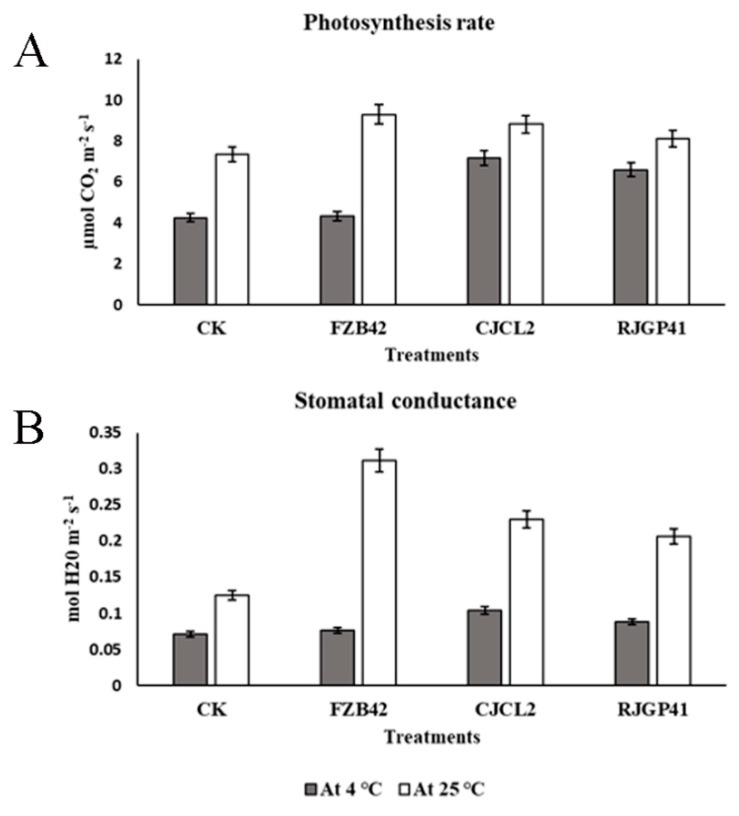
The photosynthesize rate of wheat plants under treatment of different bacterial strains (**A**) Stomatal conductance under the influence of various bacterial treatments in wheat plants (**B**). The error bars represent the standard deviation of the mean (*n* = 5). The significant difference among the treatments was observed at *p* ≤ 0.05 by using Tukey’s HSD test. The experiment was repeated thrice.

The photosynthetic activity and stomatal conductance measured for wheat plants under cold stress and at regular temperature under four bacterial treatments showed that photosynthesis rate was significantly enhanced in plants treated with *Bacillus* sp. CJCL2 under cold stress (Figure 10). The next significant values obtained for photosynthesis rate were given by plants treated with RJGP41 strain. The stomatal conductance was also enhanced in plants treated with CJCL2 strain under cold stress with comparison to other treatments. At regular growth temperature, *Bacillus* strain FZB42 showed the most significant enhancement in results for photosynthetic activity as well as stomatal conductance in comparison to control plants.

### 3.10. In Planta Growth Promotion Under Cold Stress by Inoculated Bacteria

**Figure 11:**
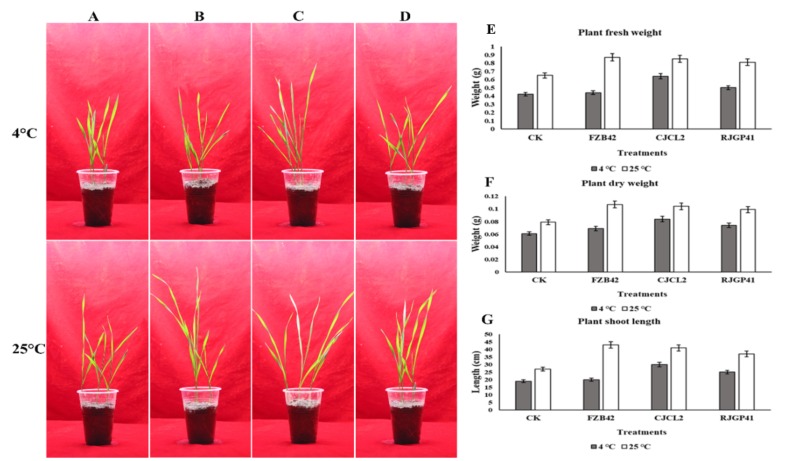
Un-inoculated wheat plants grown at optimum temperature and under cold stress (**A**) Wheat plants inoculated with Bacillus sp. FZB42 (B) Wheat plants inoculated with Bacillus sp. CJCL2 (**C**) Wheat plants inoculated with Bacillus sp. RJGP41 (**D**) Fresh weight of wheat plants grown under cold stress and at regular temperature with different bacterial treatments (**E**) Dry weight of wheat plants (**F**) Total length of wheat shoots grown under cold stress and at regular temperature with different bacterial treatments (**G**). The error bars on the graphs indicate the standard deviation of the mean (*n* = 3). The significant difference between the treatments was observed by using Tukey’s HSD test at *p* ≤ 0.05 and the experiment was repeated three times.

The improvement in wheat plants under cold stress was observed by using the seedlings already grown for 7 days and were transplanted into pots with all respective treatments. Significant improvements were observed for plant growth related parameters as well as root morphological parameters. For plants grown under cold stress, the *Bacillus* sp. CJCL2 showed most significant results for growth enhancement i.e., nearly one-fold increase in plant fresh/dry weight and shoot length in comparison to un-inoculated control plants (Figure 11). *Bacillus* sp. RJGP41 inoculated plants also showed significant growth enhancement whereas FZB42 treated plants could not show significant results under cold stress. Whereas at optimum growth temperature i.e., 25 °C, All the *Bacillus* strains showed significant enhancement in plant fresh/dry weight and shoot length in wheat plants in comparison to control plants with not much significant difference among all applied *Bacillus* strains. 

**Figure 12:**
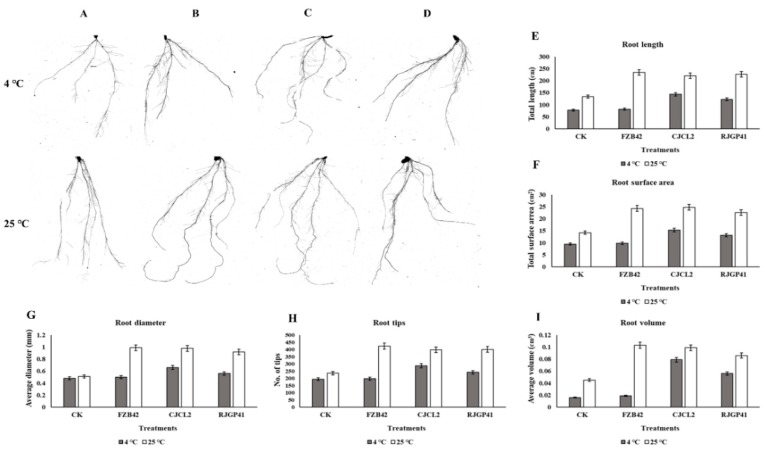
The rhizoscanning image of roots of un-inoculated wheat plants grown at 4 °C and 25 °C (**A**), The rhizoscanning image of roots treated with FZB42 (**B**), The rhizoscanning image of roots treated with CJCL2 (**C**), The rhizoscanning image of roots treated with RJGP41 (**D**) Root morphology parameters such as Total root length (cm) of wheat plants inoculated with selected bacterial strains and grown at 4 and 25 °C (**E**), The total surface area (cm^2^) of roots of wheat (**F**), the diameter of the roots (mm) (**G**), number of root tips for each treatment (**H**) the total root volume (cm^3^) (**I**) are comparatively represented by graphs. The error bars shown on the graphs represent standard deviation of the mean (*n* = 5). The significance among different treatments was observed at *p* ≤ 0.05 by using Tukey’s HSD test. The experiment was performed in triplicate with similar results.

The root morphological studies demonstrated that the parameters such as root length and root volume showed 2–3 fold increase in plants inoculated with Bacillus sp. CJCL2 in comparison to un-inoculated control under cold stress. root diameter, Root surface area and number. of tips were also found to be significantly enhanced in case of plants treated with Bacillus sp. CJCL2 under cold stress followed by the plant inoculated with RJGP41 strain (Figure 12). In comparison, the plants inoculated with all three Bacillus strains showed significant improvement in root morphological parameters at regular temperature i.e., 25 °C. 

### 3.11. Expression Profiling of Growth Related Genes in Plants Under Cold Stress

**Figure 13:**
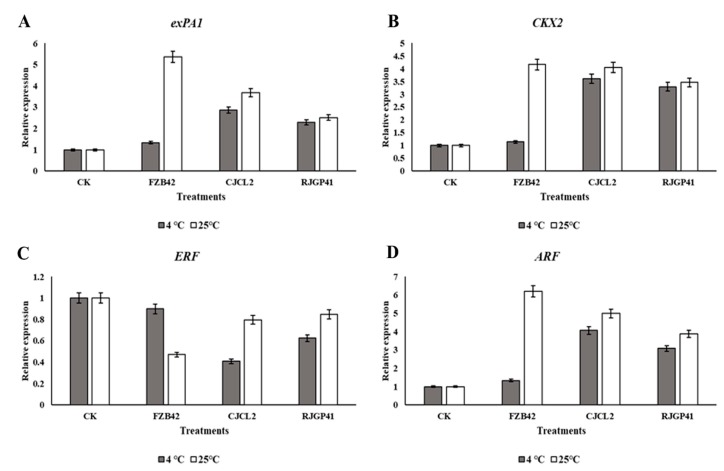
Relative expression level of expansin (*exPA1*) in wheat plants inoculated with different *Bacillus* strains grown under cold stress and at regular temperature (**A**), Relative expression level cytokinin encoding gene (*CKX2*) (**B**) Relative expression level of ethylene encoding gene (*ERF*) (**C**) Relative expression level Auxin (*ARF*) encoding gene (**D**). The error bars on the graphs indicates the standard deviation of the mean (*n* = 3). The significant difference between the treatments was observed at *p* ≤ 0.05 by using Tukey’s HSD test. The expression analysis was done three times with similar results.

The expression analysis of genes involved in plant growth promotion showed an upsurge in plants treated with *Bacillus* spp. Under cold stress i.e., 4 °C, the plants treated with CJCL2 strain showed maximum up-regulation in gene expression of Expansin (*expA1*), Cytokinin (*CKX2*) and Auxin (*ARF*) followed by the plants treated with RJGP41 (Figure 13). The expression of ethylene encoding gene *ERF* was significantly down-regulated in plants grown under cold stress and inoculated with *Bacillus* sp. CJCL2. The plants grown at 25 °C showed five, four and six fold up-regulation of expression in *expA1, CKX2* and *ARF* genes respectively under the treatment of FZB42 strain. The other two inoculated *Bacillus* strains also caused significant increase in expression of these plant growth promoting genes. Significant down-regulation of *ERF* gene was observed in all inoculated plants with minimum expression of ethylene encoding gene was observed in plants treated with FZB42 strain at optimum growth temperature of wheat.

## 4. Discussion

The abiotic stresses mount great threat to cereal crops production causing food security issues [51]. In addition to plant’s own defensive mechanism, improved crops have been developed through molecular breeding and genetic engineering for combating abiotic stress. Due to the reason that these methods are more labor intensive and time consuming, a sustainable and eco-friendly approach is the use of Plant Growth Promoting Rhizobacteria (PGPR) to improve the stress tolerance in plants [52]. The abiotic stress tolerance in plants can be induced by inoculating them with PGP bacterial species [53,54]. The *Bacillus* spp. CJCL2 and RJGP41 isolated from Qinghai-Tibetan plateau also known as the third pole of the world; were able to grow well as indicated by their growth curve at 14 °C, 10 °C and 4 °C at different time intervals for up to 96 h. Another well reported PGP strain the *B. velezensis* FZB42 strain, was also used. This strain showed slight growth at 14 °C but it was not able to grow at 4 °C. All *Bacillus* bacteria under study were able to grow well at regular growth temperatures i.e., 25 °C and 37 °C. 

In the current study, we tried to explore the genetic features in all three strains that could be responsible for imparting cold stress tolerance in these bacteria. Our results showed that the genes linked to membrane transport, fatty acid and lipid metabolism, regulation and cell signaling and those related to combat stress were detected in cold tolerant strains CJCL2 and RJGP41 and absent in temperate strain FZB42. All of these genetic features have a major role in cold stress tolerance in bacteria as reported by Allen et al. [55]. The molecular detection of predicted genes showed that the cold tolerant *Bacillus* strains CJCL2 and RJGP41 contain a plethora of genes to withstand cold stress. The genes related to anti-oxidant enzymes *SodA*, *trxA*, *KatA* and *perR* were all present in cold tolerant bacteria. The presence of these genes enables these *Bacillus* bacteria to overcome the cellular damage due to free oxy-radicals [20]. The genes *desR*, *desK*, *ResD* and *DegS*, *dpsU20* corresponding to signal transduction pathways and general stress response respectively were detected in cold tolerant strains. The detection of genes involved in signal transduction pathways suggests that our *Bacillus* bacteria have the ability to sense and respond to various stresses and respond to such environmental shocks at a very primary level [19]. The genes corresponding to osmotic stress regulation i.e., *ohrR* and *OpuAC* were also found in cold tolerant *Bacillus* highlighting their potential to maintain osmotic balance in the cell by possessing glycine betaine which is a major osmo-protectant and a constitutive product of *OpuAC* gene [56]. The presence of *ComA* gene in our cold tolerant bacteria is also essential as it is a major two-component response quorum sensing regulator as reported in the previous study [57].

Time frame study for expression profiling of the genes related to cold stress tolerance carried out in all three *Bacillus* strains under study showed an upward surge in the expression of these genes in psychrophilic *Bacillus* spp. CJCL2 and RJGP41 which could be a vital reason behind their potential to survive at lower temperature. The lower expression of all these genes could be a reason why FZB42 cannot survive at lower temperature. A linear four–five fold increase in the expression of cold shock proteins (*CspB*, *CspC*, *CspD*) observed in strains CJCL2 and RJGP41 at each time interval up to 96 h. This result corresponds to the results of Kaan et al. [58] and this could be the basis for the adaptability of these strains to colder environment as these proteins can trigger modifications in RNA and also stabilizes the secondary structure of nucleotides resulting in improved functions of cellular constituents [59]. The higher expression of response quorum sensing regulator gene *ComA* also aids cold tolerant strains to survive at lower temperatures as it acts as transcriptional activator for many important physiological responses in bacteria as reported by [60] but to the best of our knowledge this study is the first report of this gene being upregulated under cold stress. This gene also showed slight increase in expression for temperate strain FZB42 at 96 h and we assume this gene controls multiple response factors in bacteria and due to extreme stress for this bacterium at 4 °C this gene might have shown slightly increased expression. Higher expression of two-component signal transduction pathway genes *DesK* and *DesR* at 72 h and 96 h in CJCL2 and RJGP41 respectively indicate their ability to sense and respond quickly to abiotic stress or the changing environment as referred by Sun et al. as well [61]. The gene *OhrR* and glycine betaine encoding gene *OpuAC* are involved in osmotic stress regulation and are important osmo-protectant. *OhrR* genes showed maximum upsurge of expression (3.5 fold) in CJCL2 at 72 h post inoculation and almost similar expression was observed in RJGP41 as well whereas *OpuAC* gene showed a linear increase in expression in both of these strains up to 96 h with almost a five-fold upsurge of expression in CJCL2. Our results are supported by the fact that these transcription factors are activated upon abiotic stress as reported Helmann et al. [62]. The detection and expression analysis of all these genes suggested that psychrophilic *Bacillus* strains used in this study possess a complete range of genetic features enabling them to survive under cold stress.

The Reactive Oxygen Species (ROS) are the potent free oxy-radicals that can be produced as a result of biotic/abiotic stress and lower temperatures are reported to increase ROS concentration and stability [20], and if these species are produced in abundance they can disrupt the cellular functions and could eventually lead to cell death [63]. When we grew all three *Bacillus* bacteria under cold stress for 96 h, the temperate strain produced highest ROS as observed by green fluorescence emitted by the bacterial cells indicating higher cellular disruption in this strain whereas least number of ROS labeled cells were observed for strains CJCL2 and RJGP41 indicating that these bacteria can endure cold stress. Another important feature possessed by bacteria to withstand cold stress is biofilm formation. It is an accumulation of bacteria in a self-secreted matrix enabling the bacterial communities to survive and produce their metabolites in harsh environmental conditions [64]. Best biofilm structure was also observed in CJCL2 under cold stress at 48 h and 96 h post inoculation in LBGM media whereas FZB42 produced best biofilm structures at both time intervals at optimal temperature for bacterial growth. There have been some studies suggesting distortion in bacterial biofilm structures under stress [65] but there are very scarce evidences showing development of biofilm at low temperature as reported by [66] where a food-related pathogen *L. monocytogenes* could form biofilm at low temperatures. We assume that the biofilm forming ability of psychrophilic PGPR *Bacillus* used in this study is a unique trait enabling them to survive and produce important metabolites under cold stress. This ability is linked with the potential of inoculated bacteria to colonize plant roots and aid the plants in alleviating cold stress.

The ability of bacteria to alleviate different abiotic stresses such as salinity, drought, and oxidative stress in plants has been reported previously [29,67]. To the best of our knowledge, the present study is the first report for the alleviation of cold stress in wheat plants by the inoculation of psychrophilic *Bacillus* spp. CJCL2 and RJGP41 isolated from the Qinghai-Tibetan plateau. We report that these strains can induce regulation of the stress response parameters such as abscisic acid (ABA), proline accumulation, and lipid peroxidation in winter wheat (cultivar Jimai22) when grown at 4 °C. The temperate PGPR strain FZB42 could not influence these parameters under cold stress but was able to decrease the ABA levels when plants were grown at 25 °C due to the fact that this strain is a potent biocontrol and growth promoting agent under optimum growth conditions. ABA levels are increased in plants during osmotic stress and inoculated *Bacillus* spp. can lower the ABA levels in plants to alleviate drought or salinity stress as reported by [68], and these results are in accordance to our study showing a decrease in plant ABA levels under cold stress induced by the inoculated *Bacillus* spp. CJCL2 and RJGP41. Proline accumulation and reduced malondialdehyde (MDA) levels are vital indicators in plants to withstand different abiotic stresses as proline acts as an osmolyte and osmoprotectant [69] whereas increased MDA levels show oxidation and damage of membrane lipids resulting in distortion of cellular integrity in plants [70]. The results of the present study indicate that wheat plants exposed to cold stress had an increased level of accumulated proline and decreased MDA level upon inoculation with cold tolerant bacteria and our results conform to those of [47] as they also reported the same when they inoculated *Bacillus amyloliquefaciens* in rice to alleviate drought and salinity stress. We also report the corresponding gene expression levels of all these stress response parameters and the same pattern in expression levels also strengthen our claim of positive regulation toward enduring cold stress in plants by the inoculation of psychrophilic bacteria. In addition to these stress responses, our study is the first report for improvement in photosynthetic efficiency and stomatal conductance of wheat plants by inoculated *Bacillus* spp. CJCL2 and RJGP41 under cold stress i.e., 4 °C. There have been some studies suggesting the improvement in these parameters by inoculated bacteria [49] but no previous study has indicated such results under cold stress in wheat plants.

The application of PGPR to enhance plant growth has been widely reported [71]. There have been reports suggesting plant growth enhancement by PGPR under various abiotic stress such as cold stress has already been reported by using *Bacillus* spp. in previous study of our lab [72]. PGPR can enhance plant growth as reported by [73] under salt stress. Another study by [74] indicate the growth promotion in plants by *Pseudomonas* sp. under elevated temperatures. Similarly [75] has reported the pea plant growth promotion by inoculating *Pseudomonas* spp. under drought stress. We hereby report the improvement in wheat plant growth by *Bacillus* spp. CJCL2 and RJGP41 under cold stress through seed inoculation. The seed priming and seedling inoculation could lead to better colonization of these *Bacillus* and result in plant growth enhancement under stressed environments. We also observed the best results for all seedling and plant growth promotion parameters at optimum growth temperature by all three *Bacillus* strains CJCL2, RJGP41 and FZB42 but this study is the first report on *B. velezensis* strain FZB42 to not have any significant influence on plant growth under cold stress. *Pseudomonas* and *Bacillus* strains are well documented to have positive effect on vigor index and root morphological parameters of seedlings and young plants [43,44] but in present study we report significant improvement in vigor index and root morphological parameters of wheat seedlings as well as wheat plants under cold stress by the inoculation of psychrophilic *Bacillus* CJCL2 and RJGP41. Under cold stress, fresh/dry weight and shoot length of wheat plants inoculated with these bacteria also showed significant increase as compared to control plants. The PGPR used in this study possess the genes for ACC deaminase (*acds*) involved in catalysis of ACC to ammonia and alpha ketobutyrate which results in decreased ethylene levels and increase plant growth [42], glucose dehydrogenase (*gdh*) which is involved in production of organic acids [76] and pyrroloquinoline quinone (*pqqE*) which is major component of *PQQE* operon and is involved in mineral solubilization. The inoculated strains CJCL2 and RJGP41 were also able to upregulate the expression levels of genes responsible for producing important plant growth hormones such cytokinin (*CKX2*), auxin (*ARF*), alpha expansin (*exPA1*), and ethylene (*ERF*) under cold stress whereas all three *Bacillus* strains showed up-regulation in the genes of these phytohormones at optimum temperature for wheat growth. Many studies have emphasized the importance of these phytohormones in plant growth promotion [77,78,79,80,81]. The phytohormone i.e., cytokinin, auxin and expansin related genes is also up-regulated by the inoculation of PGPR as reported by [82] and the ethylene gene is reported to be down-regulated [81], The expression pattern also conforms to our results as well But to the best of our knowledge, the present study is the first report of transcriptional regulation of these phytohormone genes in wheat plants under cold stress induced by inoculated *Bacillus* strains CJCL2 and RJGP41. We assume that improvement in plant growth by the inoculated bacteria under cold stress is an accumulative effect of their ability to reduce stress in plants, possessing genes to promote plant growth and regulating the expression of important plant phytohormones.

The significance of this study is highlighted by the fact that we have tried to explore the genetic features of potential psychrophilic Bacillus spp. CJCL2 and RJGP41 and temperate PGPR FZB42 in order to detect and study the expression of the genes which could impart cold tolerance in these microbes enabling them to perform their metabolic and physiological functions efficiently under cold stress. Due to the potential of these bacteria to alleviate cold stress and promote plant growth by modulating phytohormones at low temperature, it is highly desirable to further investigate these psychrophilic bacteria to use them in the form of different bio-formulations and develop bio-fertilizers for better agricultural production in extreme environments.

## Supplementary Materials

The following are available online at https://www.mdpi.com/2076-2607/7/9/337/s1.
