# Supplementary material for: Genetic Screening and Expression Analysis of Psychrophilic Bacillus spp. Reveal Their Potential to Alleviate Cold Stress and Modulate Phytohormones in Wheat
Source: Microorganisms. 2019 Sep 10;7(9):337. doi: 10.3390/microorganisms7090337 (PMC6780275; doi:10.3390/microorganisms7090337)
Supplement: Supplementary file 1 [file microorganisms-07-00337-s001.pdf]

Table S1: DNA primers used in this study for detection of genes by PCR

No	Oligo Name	Sequence 5/ to 3/
1	*DesR* (F)	CGCCAGCCTGATGTATGTATTA
	DesR (R)	CTGAGTTCCTGAGCGATTTCTT
2	*SodA* (F)	CACTGCTCTTGCCAACAAATC
	SodA (R)	CCACTTCGTCCCAGTTTACAA
3	*DesK* (F)	GCCTCCATTACTCTGTTCAGTT
	DesK (R)	TGAGATATTCTCGGGCCATTTC
4	*trxA* (F)	CAGACTTCTGGGCTCCTTG
	trxA (R)	TTGGTTTGAAGCCGACTGA
5	*Res D* (F)	GTAGTTGATGACGAAGCCAGAA
	Res D (R)	CGGTGAGCATCATGGTCTATT
6	*ohrR* (F)	CCGCTGCTTGATAAGCTGAATA
	ohrR (R)	CCCAGAATTGTCCCTGGAATG
7	*DegS* (F)	GAGGAAATCCGCAATGCTTATG
	DegS (R)	CCTTCAGGTCGAACCCTTTAC
8	*ComA* (F)	ACAGTCCTCAGTTTAGCGTTTAT
	ComA (R)	AATCAGCGGCCACAAGAA
9	*dpsU20* (F)	GCAGTGAACAAACAAGTAGCC
	dpsU20 (R)	TGGATCGCAAGAAGCATGT
10	*OpuAC* (F)	AGCGCAGAGGAAATCCATAC
	OpuAC (R)	CCCATCTTCACACCAGTCATAC
11	*KatA* (F)	ACCGTTGTTCATCCGTTTCT
	KatA (R)	CGAAGCTGTTAGGCTCGTAAT
12	*PerR* (F)	CGCATTACTCCTCAACGTCAT
	PerR (R)	GCAGACGCCGTAAATTTCTAAAC
13	*acds* (F)	ATGAAYCTSCARCGHTTY
	acds (R)	TYARCCGTYSCGRAARRT
14	*gdh* (F)	TCTACAGGTTTGGGAAAATCAATGGCG
	gdh (R)	TTATCCGCGTCCTGCTTGGAATGA
15	*pqqE* (F)	GARCTGACYTAYCGCTGYCC
	pqqE (R)	TSAGSAKRARSGCCTGR

Table 2. qPCR primers for expression analysis used in this study.

No	Oligo Name	Sequence 5/ to 3/
1	*cspB* (F)	AGGTTTCGGATTCATCGAAGTAG
	cspB (R)	TAGTAACGTTAGCAGCTTGTGG
2	*cspC* (F)	GGAACAAGGTACAGTTAAATGGTTT
	cspC (R)	CTTGCTCAACGTCAAACGATAC
3	*cspD* (F)	TGAAGTTGAAGGCGGAGAC
	cspD (R)	ATTAGAAGCTTGAGGTCCACG
4	*comA* (F)	CTCACACCTAGAGAATGCCTG
	comA (R)	CTGTATTCAATGGACCGCTTG
5	*desK* (F)	GAGGATGCAAATGAACGGATTG
	desK (R)	TGCTTGTTCGGGATCTTTGTA
6	*desR* (F)	CCGGTTACTTTCAGAGAGCTATTA
	desR (R)	GAGGGTTAGCTTCGCTGTATAA
7	*ohrR* (F)	CGGGAGATGACAAAGCAATACA
	ohrR (R)	TTCGTTTAAGCATCGGAGTGAG
8	*OpuAC* (F)	AGCGCAGAGGAAATCCATAC
	OpuAC (R)	GACGTACTCAGCAGCAACTT
9	*ABARE* (F)	TTACACCGTGGAGCTTGAAG
	ABARE (R)	TTCACGTTCTCCTTGGACTG
10	*4-HNE* (F)	CAAGTACCACTTCGACCTGAG

	4-HNE (R)	CCTCTTGAACTGGATGTCGATG
11	*P5CS* (F)	GATTCTCCGATGGTGCTCGT
	P5CS (R)	TTCAACACCCACAGGTCCAC
12	*ExpA1* (F)	TTGCTGATACAAGGAAGCTCTC
	ExpA1 (R)	GTCGTAGAAGGTGGCATGG
13	*CKX2* (F)	CGGGATAGGGTACAAGGAGTA
	CKX2 (R)	GTACTTGTCCTTCATCTCCACG
14	*ERF* (F)	TGCCCTTCGATCTGAACATG
	ERF (R)	TCCCACGAATCCTTGCTATTC
15	*ARF* (F)	AGATGTTCAGCACCTTCACC
	ARF (R)	CAGTCTCCATCCTTGTCTTCG
